# Case of Carbapenem-Resistant *Salmonella* Typhi Infection, Pakistan, 2022

**DOI:** 10.3201/eid2911.230499

**Published:** 2023-11

**Authors:** Summiya Nizamuddin, Ejaz Ahmed Khan, Marie Anne Chattaway, Gauri Godbole

**Affiliations:** Shaukat Khanum Memorial Cancer Hospital and Research Centre, Lahore, Pakistan (S. Nizamuddin);; Shifa International Hospital at Shifa Tameer-e-Millat University, Islamabad, Pakistan (E.A. Khan);; UK Health Security Agency, London, United Kingdom (M.A. Chattaway, G. Godbole)

**Keywords:** Salmonella Typhi, Salmonella enteria serovar Typhi, therapy, diagnosis, antimicrobial resistance, carbapenem resistance, typhoid fever, bacteria, Pakistan

## Abstract

*Salmonella* Typhi infection in a patient in Pakistan initially responded to standard treatment but failed to respond to subsequent treatment. The first strain was susceptible to carbapenems and azithromycin; subsequent strains harbored the NDM-5 gene. Treatment with a combination of intravenous meropenem and colistin was successful. Carbapenem-resistant *Salmonella* Typhi emergence will hinder treatment.

Extensively drug-resistant (XDR) *Salmonella* was first reported in Sindh, Pakistan, in 2016 (*1*). Since then, several districts have reported cases caused by multiple XDR *S. enterica* serovar Typhi variants belonging to clade H58 and carrying multiple novel genomic integrations of the extended-spectrum β-lactamase gene (*2*,*3*). Most cases are managed with meropenem and azithromycin, and most national guidelines recommend those drugs for treating cases of *Salmonella* Typhi in Pakistan (*4*,*5*). We report a case of carbapenem-resistant *Salmonella* Typhi infection that required treatment with a last-resort antimicrobial drug.

A 7-year-old girl in Peshawar, Pakistan, visited a government hospital in July 2022 because of fever, chills, rigors, and urinary signs/symptoms. She had Down syndrome with congenital heart disease, including moderate atrial septal defect, large inlet ventricular septal defect, small patent ductus arteriosis defects, ventricular hypertrophy, and severe pulmonary hypertension; and she had a history of reoccurring lower respiratory tract infections, which often required hospitalization and treatment with antimicrobial drugs. Macrolides and carbapenems had been previously prescribed. Specific information regarding previous hospital admissions was not available. The child had received routine childhood vaccinations but not typhoid vaccine.

The patient was hospitalized for suspected enteric fever. At admission, leukocyte count was 7.6 × 10^9^ cells/L and C-reactive protein (CRP) level was 120 mg/dL. Initial empiric treatment was ceftriaxone, but treatment was modified after blood cultures indicated XDR *Salmonella* Typhi, resistant to ampicillin, third-generation cephalosporins, fluoroquinolones, chloramphenicol, and cotrimoxazole but susceptible to azithromycin and meropenem. Treatment was switched to intravenous meropenem (300 mg 3×/d) and oral azithromycin (200 mg/5 mL 1×/d) for 10 days. The strain from the culture was not saved and was unavailable for additional testing. The patient responded positively to treatment, indicated by decreased CRP levels (7 mg/dL at treatment completion). No clearance blood cultures were obtained before discharge.

One month later, fever and decreased appetite developed; blood was collected at a private laboratory, and cultures were requested. We monitored the blood culture on a BACT/ALERT VIRTUO automated system (bioMérieux, https://www.biomerieux.com). When the bottle was flagged as positive for gram-negative rods, we subcultured onto chocolate, blood, and MacConkey agar plates; after 24 hours, oxidase-negative, non–lactose fermenters were identified. *Salmonella* Typhi was identified on the API 20E and Vitek MS (bioMérieux) systems. Antimicrobial susceptibility testing was initially performed via the disc diffusion Kirby-Bauer method, according to Clinical Laboratory Standards Institute guidelines (https://www.clsi.org). The isolate was resistant to ampicillin, third-generation cephalosporins, fluoroquinolones, chloramphenicol, cotrimoxazole, azithromycin, and meropenem. We further confirmed susceptibility results on the Vitek 2 system. MICs for meropenem and azithromycin were >32 μg/mL. We confirmed results by using bioMérieux Etest strips. MIC for colistin, determined by disk-elution method, was 1 μg/mL.

The patient’s parents were informed about the panresistant nature of the isolate, and the patient was referred to a private hospital for further management. At admission, she had fever, anorexia, and abdominal pain but was not in distress and was active and alert (Glasgow Coma Scale score 15/15). She had dysmorphic features consistent with Down syndrome. Her chest was bilaterally clear, and respiratory rate was within reference limits. Her abdomen was soft, nontender, and nondistended, with no hepatosplenomegaly. On auscultation of the cardiovascular system, fixed splitting of S2 and grade 3 holosystolic murmur were audible. The patient’s extremities exhibited no skin lesions and were well perfused. Initial investigations indicated leukocyte count 7,600 cells/μL, hemoglobin 14.4 g/dL, platelets 61,000 cells/μL, and CRP 11.86 mg/L. Chest radiographs revealed mild cardiomediastinal magnification and congestive changes along with right hilar congestion but no consolidation, collapse, or definite pneumothorax. Costophrenic angles were intact.

After admission, treatment with intravenous meropenem (700 mg 3×/d infused over 2 h) and intravenous colistin (40 mg 2×/d) was initiated. The patient was closely monitored and became afebrile on day 3 of therapy. A 2-dimensional echocardiogram showed no vegetations. Results of repeat blood cultures and fecal cultures were negative. The patient was discharged after completing 11 days of treatment. At a 2-week follow-up visit, she was back to her usual state of health; typhoid vaccination 2 weeks later was recommended.

The isolate obtained during relapse was sent to the UK Gastrointestinal Bacteria Reference Unit, where we confirmed *Salmonella* Typhi by PCR (*6*) and cultured it on blood and MacConkey agar plates to ensure purity. We isolated 2 distinct morphologic variants: variant 1 (large colony, isolate 1790097) and variant 2 (small colony, isolate 1790125) (Figure). Each variant underwent whole-genome sequencing for single-nucleotide polymorphism typing, core-genome multilocus sequence typing, and antimicrobial resistance determination (*7*,*8*). We analyzed phylogeny of both strains in the context of *Salmonella* Typhi reported in England in 2016–2019 (Appendix Figure) and visualized them on ITOL (*9*). We performed MIC testing by using a Thermo Scientific sensititre broth microdilution system (https://www.thermofisher.com) on EUVSEC2 and EUVSEC3 plates and confirmed carbapenemase production by Liofilchem metallo-β-lactamase Etest (http://www.liofilcheminc.com). Breakpoints and screening concentration criteria used for interpretation were recommended by the European Committee on Antimicrobial Susceptibility Testing (*10*).

**Figure:**
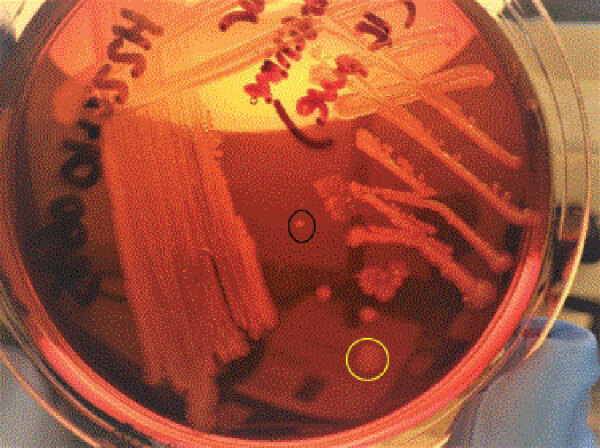
*Salmonella* Typhi colony on MacConkey agar, isolated from patient during clinical relapse of carbapenem-resistant *Salmonella* Typhi infection, Pakistan, 2022. Variant 1, circled in yellow, is a large gray dull colony. Variant 2, circled in black, is a small whitish shiny colony.

Variants 1 and 2 were XDR *Salmonella* Typhi, resistant to azithromycin and carbapenems; variant 2 had additional antimicrobial resistance determinants (Table). Testing with the metallo-β-lactamase Etest indicated that both variants were carbapenemase producers.

**Table:** MICs and antimicrobial drug resistance determinants of *Salmonella* Typhi isolated from patient during clinical disease relapse of carbapenem-resistant *Salmonella* Typhi infection, Pakistan, 2022*

Antimicrobial drug	1790097 (variant 1)			1790125 (variant 2)	
	Gene/ mutation	MIC, μg/L		Gene/mutation	MIC, μg/L
Ampicillin	*bla*_NDM-5_, *bla*_TEM-1_	32		*bla*_NDM-5_, *bla*_TEM-1_, ***bla*_CTX-M-15_**	32
Azithromycin	*mph*A	64		*mph*A	64
Cefoxitin	*bla*_NDM-5_	64		*bla*_NDM-5_	64
Tetracycline	*tet*A	32		*tet*A	32
Tigecycline	NA	0.25		NA	0.25
Ertapenem	*bla*_NDM-5_	2		*bla*_NDM-5_	2
Imipenem	*bla*_NDM-5_	16		*bla*_NDM-5_	16
Meropenem	*bla*_NDM-5_	16		*bla*_NDM-5_	16
Cefotaxime	*bla*_NDM-5_	64		*bla*_NDM-5_, ***bla*_CTX-M-15_**	64
Ceftazidime	*bla*_NDM-5_	128		*bla*_NDM-5_, ***bla*_CTX-M-15_**	128
Cefepime	*bla*_NDM-5_	32		*bla*_NDM-5_, ***bla*_CTX-M-15_**	32
Nalidixic acid	*gyrA* [83:S-F]	64		*gyrA* [83:S-F], ***qnrS1***	64
Ciprofloxacin	*gyrA* [83:S-F]	1		*gyrA* [83:S-F], ***qnrS1***	**8**
Chloramphenicol	NA	8		***catA***	**64**
Trimethoprim	*dfrA-27*, *dfrA-7*	16		*dfrA-27*, *dfrA-7*	16
Sulfonamide	*sul-1*	512		*sul-1*	512
Amikacin	*aac(6')-Ib-cr, aac(6')-Ia*	<4		*aac(6')-Ib-cr*, *aac(6')-Ia*	**8**
Gentamicin	*aac(6')-Ib-cr*, *aadA-16*, *aac(6')-Iy*	<0.5		*aac(6')-Ib-cr*, *aadA-16*, *aac(6')-Iy*	<0.5
Temocillin	*bla*_NDM-5_	128		*bla*_NDM-5_	128
Colistin	NA	1		NA	1

*Boldface indicates additional genes detected and increased MIC values for variant 2. NA, not applicable.

The most likely explanation for the patient’s relapse, with *Salmonella* Typhi resistant to carbapenems and azithromycin resulting from acquisition of new resistance determinants *bla*_NDM-5_ and *mphA*, is multiple genetic mutations acquired via a mobile transmissible element such as a plasmid from gut microbiota. Previous receipt of carbapenems and azithromycin for multiple respiratory infections likely led to harboring and selection pressure for carbapenemase-producing strains. Another possibility is a suboptimal host immune response because of Down syndrome, although the patient had not undergone an immunology assessment. Expansion of antimicrobial-resistant strains will make such infections extremely difficult to treat.

Appendix Additional information for carbapenem-resistant *Salmonella* Typhi isolate from a patient in Pakistan, 2022.
