# Supplementary material for: Case of Carbapenem-Resistant Salmonella Typhi Infection, Pakistan, 2022
Source: Emerg Infect Dis. 2023 Nov;29(11):2395–7. doi: 10.3201/eid2911.230499 (PMC10617351; doi:10.3201/eid2911.230499)
Supplement: Appendix — Additional information for carbapenem-resistant Salmonella Typhi isolate from a patient in Pakistan, 2022. [file 23-0499-Techapp-s1.pdf]

Article DOI: https://doi.org/10.3201/eid2911.230499

Case of Carbapenem-Resistant *Salmonella* Typhi Infection, Pakistan, 2022

Appendix

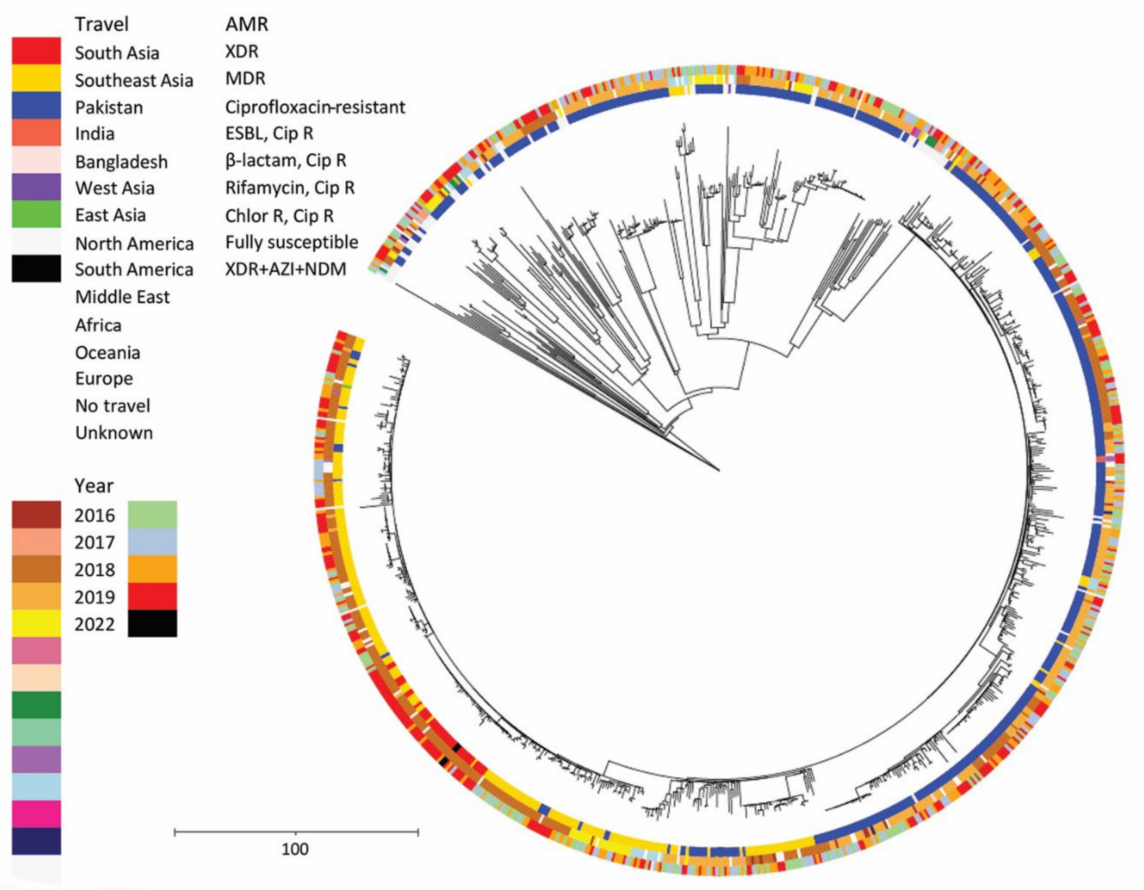

Appendix Figure. Phylogeny of the *Salmonella* Typhi in England, 2016–2019, including imported ESBL producing strains (*bla* $_{CTX\text{-}M\text{-}15}$) and the case for Pakistan. Phylogenetic tree of cgMLST analysis of S. Typhi strains generated using the neighbor-joining method and mapped against three categories: inner ring, AMR (XDR, extensively drug resistant strains; MDR, multidrug resistant strains; Cip R, ciprofloxacin-resistant strains; ESBL, extended-spectrum β-lactamase producing strains; Chlor R, chloramphenicol-resistant strains, XDR+AZI+NDM, XDR with azithromycin and carbapenemase resistance); middle ring, travel; outer ring, year. Both pan-resistant strains from 2022 (black) fall into the HC5_1452 XDR sub-cluster associated with travel to Pakistan.
